# Pre-donation deferral of blood donors in South Indian set-up: An analysis

**DOI:** 10.4103/0973-6247.67037

**Published:** 2010-07

**Authors:** P. Sundar, S. K. Sangeetha, D. M. Seema, P. Marimuthu, N. Shivanna

**Affiliations:** 1 *Department of Neuropathology, National Institute of Mental Health and Neurosciences, Bangalore - 560 029, India*; 2 *Transfusion Medicine Centre, National Institute of Mental Health and Neurosciences, Bangalore - 560 029, India*; 3 *Clinical Pathology Lab, National Institute of Mental Health and Neurosciences, Bangalore - 560 029, India*; 4 *Department of Biostatistics, National Institute of Mental Health and Neurosciences, Bangalore - 560 029, India*

**Keywords:** Blood donor, deferral, permanent, temporary

## Abstract

**Background:**

It is well known that quite a large number of apparently healthy donors are not able to donate blood successfully because of varied reasons.

**Aim:**

We want to analyze the rate and various reasons for deferrals.

**Materials and Methods:**

A retrospective analysis of records of the donors, for 3 years, from January 2005 to December 2007 was done, in order to find out the rate and causes of deferral in four categories of age groups, both in male and female, in our Transfusion Medicine Centre, Bangalore, India.

**Result:**

There were 16,706 donors, of which 976 donors were deferred (5.84%) for various reasons. Of the 16,706 donors registered for donation, females constituted only 11.27%. And deferral rate was about five times more for female (19.85%) compared to male (4.06%). The three most common reasons for deferral in female were low hemoglobin levels, low body weight, and hypotension. The deferral rate was higher in the age group of 18-25 years and most common cause was low hemoglobin level. In male, the three most common reasons for deferral were hypertension, under weight, and low hemoglobin levels. The deferral rate varied from 4 to 15% as reported in the literature. The most common cause of deferral in our study and in several studies available in the literature is the same.

## Introduction

Blood donor suitability criteria are based on science, informed medical opinion, and regulatory rules.[1] Blood donors are deferred for various reasons. Individuals disqualified from donating blood are known as “deferred” donors. To make blood transfusion safe for the patients many safety measures are undertaken by the blood transfusion community. Of the many safety measures, the most important is selection of blood donors. The rate and reasons of deferral differs from region to region and one center to the other. To protect blood donors and recipients, stringent donor screening criteria are necessary.[2]

## Materials and Methods

The study involved donors both voluntary and replacement who have donated blood to our center during the period January 2005 to December 2007. During which period, there were 16, 706 donors who came to donate whole blood. Of 16,706, there were 14,822 (88.72%) males and 1884 (11.27%) female donors. We collected blood from donors both at the Transfusion Medicine Center (TMC) involving both voluntary and replacement donors; and out door camps involving only voluntary donors. Approximately 90% were voluntary and 10% replacement. The majority of donors were people in and around Bangalore within a radius of 50 km. A representative group of volunteers from all over India visiting our center formed part of this donor base. The quantity of blood collected was 350 ml or 450 ml depending on the weight of the donor: 350 ml was collected from donors who weighed 45-60 kg and 450 ml from donors who weighed above 60 kg.

Each donor was selected by a medical officer based on detailed medical history and brief physical examination of donors with regard to hemoglobin, blood pressure, temperature, and pulse regularity and rate. Detailed information on the donor deferral including the cause of deferral was recorded in deferral register. Donors deferred were differentiated according to sex, age group, and whether deferral was temporary or permanent basis. Criteria laid down by director general Health Services and Drug’s Controller of India were strictly followed. Deferral by self was not considered, as it was difficult in our setup. We used statistical method to detect the rate and reason for donor deferral.

## Results

Of the 16,706 donors registered at our blood center and at various blood donation camps, 14,822 were males and 1884 females. As the figures reveal, female constituted only 11.27% of donors. The deferral rate among males was 4.06% and among females 19.85%. We have also subdivided the age group into four categories, for both male and female, to find out which category of age donated blood more and in which age group the deferral rate is high. Table 1 shows the total number of donors, number deferred, and percentage deferred both in male and female.

**Table 1:** Distribution of blood donation and deferral in by sex

	Males	Females	Total
Number of donors	14,822	1884	16,706
Number deferred	602	374	976
% deferred	4.06	19.85	5.84

As per the records the reasons for deferral are many as listed below. They are broadly differentiated into permanent and temporary. There were 820 (84%) temporary and 156 (16%) permanent deferrals out of 16,706 donors. This is shown in tables 2 and 3 by different age groups.

**Table 2:** Distribution of permanent deferral by age and sex

Causes	18-25 years		26-35 years		36-50 years		51 years and above		Total		Grand total
	M	F	M	F	M	F	M	F	M	F	
Hypertension and cardiac problems	19	0	36	1	49	2	5	0	109	3	112
H/O Icterus with or without organomegaly	10	0	2	1	1	0	0	0	13	1	14
Diabetes on insulin	1	0	0	0	3	1	3	0	7	1	08
Asthma	1	0	5	0	0	0	0	0	6	0	06
Skin disorders	1	0	2	1	1	0	0	0	4	1	05
Epilepsy	1	1	2	0	0	0	0	0	3	1	04
Chr. allergic diseases	2	0	1	0	0	0	0	0	3	0	03
Thyroid diseases	0	1	0	0	1	0	0	0	1	1	02
Renal (nephrotic syndrome)	0	1	0	0	0	0	0	0	0	1	01
Thalassemia minor	0	1	0	0	0	0	0	0	0	1	01
Total	35	4	35	3	55	3	8	0	146	10	156

**Table 3:** Distribution of temporary deferral by age

Causes	18-25 years		26-35 years		36-50 years		51 years and above		Total		Grand total
	M	F	M	F	M	F	M	F	M	F	
Anemia	26	136	7	30	11	13	1	0	45	179	224
Under weight	48	43	17	2	4	1	0	0	69	46	115
Circulatory	40	42	11	2	10	1	0	0	61	45	106
Respiratory (URI + Bronchitis)	40	12	26	2	4	1	0	1	70	16	86
Fever with bacterial, viral, fungal infection and malaria	31	8	36	0	6	1	0	1	73	10	83

**Figure 1:**
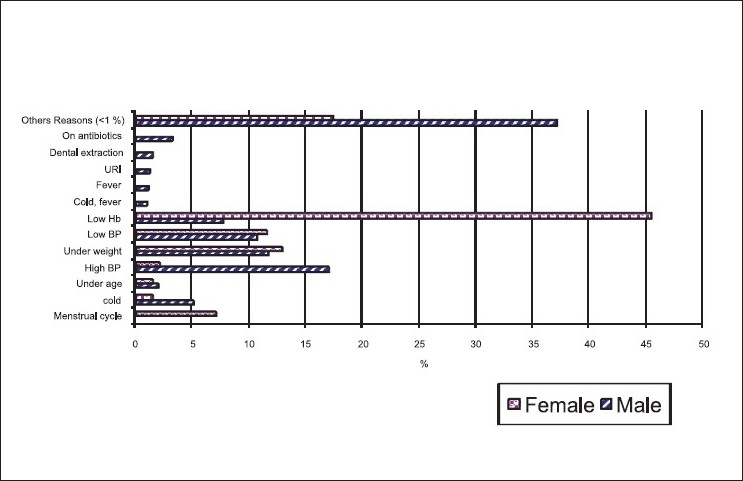
Distribution (%) of reasons for rejecting blood donors by sex (2005 to 2007)

The most common cause for deferral was anemia both in male and female donors, in our study as shown in Figure 1. The next common causes were low body weight, hypertension, hypotension, and respiratory problems such as URI and bronchitis, fever with infections, and infestations, on medications for various reasons of menstrual problems and recent dental extraction. Uncommon causes included donors with icterus with/without organomegaly, having undergone major surgeries, minor surgeries, diabetes on insulin, history of recent blood donation, asthma, skin problems like psoriasis, thyroid diseases, renal problem like nephrotic syndrome, chronic allergic diseases, hemoglobinopathy such as thalassemia minor. 18 male and 10 female donors were deferred because of underage (less than 18 years). There was only one unsuccessful phlebotomy (0.006%).

Reasons for rejecting the blood donors are shown in the Figure 1. It is observed from this graph that due to high prevalence of high anemic in the female population, the leading reason for rejecting the female donors is low Hb levels. High BP is the leading reason for rejecting the male donors. Almost the same proportion of male and female donors are rejected for low BP.

## Discussion

Deferring or rejecting potential blood donors often leaves the person with negative feeling about themselves as well as the blood banking system. But there are definite advantages of eliminating donors with possible risk of disease because despite the availability of sensitive screening tests to detect HIV infection, blood donors can be infected but test negative if they have been infected for a period of 6 weeks or less.[3] Deferring donors also protects the donors from possible adverse reactions and avoid consequent negative impact on the donor motivation.

The rate of deferral differs from region to region and sometimes in the same region and one center to another.[4] In this study the overall deferral rate was about 6% and the deferral rate was about five times higher in females compared with males i.e. one fifth of female donors were differed. The lowest reported rate of rejection was by Talonu T (4%) in Papua New Guinea[5] and higher rate (8-15%) was reported by Chaudhry,[6] Lim,[2] Blumberg,[7] Ranveet.[8]

In our study the unsuccessful phlebotomy was only 0.006%. Whereas Farrales[9] reported a higher rate of 0.5% and Custer *et al*, reported mis-collection leading to 3.8% of 1001,141 collections. Age group deferral did not show much variation and did not give significant useful information. None of the other studies attempted to classify donors into different age group. In India donors above 60 years are not allowed to donate blood. Even otherwise it is rare to have a donor voluntarily coming to donate blood in our region. And there are very few donors above 50 years constituting less than 1% in our study. Whereas in other countries there are many healthy voluntary blood donors above 60 years of age who successfully donate blood. Garry *et al*, advice elderly healthy individual to donate but to limit donations to less than five per year or donors are advised to take iron supplement regularly to preserve reasonable amount of iron reserve.[10]

Causes of deferral were many and were broadly classified into temporary and permanent. More number of deferral was in temporary constituting about 84% and permanent about 16%. Custer *et al*, report 68.5% temporary and 31.5% permanent deferral.[11] In our study permanent deferral constituted only 16%; this may be due to more number of younger donors. Most blood donor deferrals are temporary and short-term. The most common causes for temporary and short-term deferral (STTD) in female were low hemoglobin level, low body weight, and hypotension and in males low hemoglobin level and hypotension. In a study by Halperin *et al*, the three most common STTD are low hemoglobin level, colds and/or sore throats, and elevated temperature,[12] whereas that by Ranveet *et al*, under-weight, under-age, and low hemoglobin levels.[8] Hence, studies on donor deferral indicate that in each regions there would be unique sets of reasons. The effect of short-term, temporary deferral STTD on blood donor returns and subsequent blood donation is an important issue. STTD have a very negative impact on blood donor return rates and subsequent donations.[12]

In many studies it is observed that the most common cause for deferral is anemia, even in western studies. In India required hemoglobin is 125 gm/l both for male and female, for blood donation. In Canada, 2% of all blood donors do not meet minimum hemoglobin standard,[13] whereas in developing countries the number is more as pointed by this study (more than 7%).

Under permanent deferral, hypertension was the most common cause of deferral. Two Indian studies report that history of jaundice was the most common cause of deferral in Chandigarh[8] and Lucknow.[6] A large number of deferrals due to pulse irregularities or histories suggestive of potential cardiovascular problems were reported by Blumberg *et al*,[7] whereas in our study less than 1% of donors had these type of medical problems.

Having a tattoo has been associated with serological evidence of hepatitis B and C viruses, as well as HIV infection and syphilis, all these are known to be transmissible by blood transfusion. These associations are of higher magnitude for individuals having two or more tattoos unprofessionally applied and are common among drug addicts and prisoners.[14] In our region tattooing is not common and constituted less than 1% of deferral.

Donor self-deferral is valid for reducing the risk of HIV transmission through blood transfusions and its implementation should be encouraged, when recruiting blood donors.[15] In our set-up, self-deferral could not be practiced because of various reasons.

FDA recommended new travel deferrals in May 2002 to prevent potential transmission of variant CJD (vCJD).[16] We did not have donors who have traveled to UK and Europe.

Domen *et al*, indicate that shared donor deferral registries may be valuable at the local or regional level to prevent deferred blood donors from donating at other blood collection facilities.[17] In our region blood centers are not well connected and hence shared deferral registry could not be maintained.

Tomasulo *et al*, have shown that less restrictive criteria can be used for donor selection without compromising donor safety and they point out that criteria for donor deferral which are intended to exclude donors likely to suffer a “donor reaction” are based partially on untested hypothesis and tradition.[18]

In USA blood center approximately 83% of blood donors successfully donate, but 13% are rejected because of donor suitability issue. One percent is rejected for the positive test, which is often nonspecific or false positive and 2% to 4% of the phlebotomies are not successful.[19]

## Conclusion

The most common cause of temporary deferral in female donors is anemia, which is expected. However, anemia is also the most common cause among male donors contrary to the belief that men are not anemic.

Loss of units from both first time and repeat donors due to temporary deferral and loss of units from mis-collection are more common events than losses due to disease marker testing. Mis-collection does not seem to be a problem, in many settings. In our study there was only one case of mis-collection, which indicates that good technique is applied for phlebotomy by well-trained phlebotomists.

Actions to alleviate negative effects of STTD are indicated. By developing strategies to identify and rationalize donor selection criteria, the blood transfusion services should be able to decrease unnecessary deferrals. Also deferred donors should be helped to overcome their problems such that they move out from the category of Non-donors to permanent DONORS.

## References

[1] Newman B (2001). Blood donor suitability and allogenic whole blood donation. Transfus Med Rev.

[2] Lim JC, Tien SL, Ong YW (1993). Main causes of pre-donation deferral of prospective blood donors in the Singapore blood transfusion service. Ann Acad Med Singapore.

[3] Sawanpanyalert P, Uthaivoravit W, Yanai H, Limpakarnjanarat K, Mastro TD, Nelson KE (1996). Donation deferral criteria for human immunodeficiency virus positivity among blood donors in northern Thailand. Transfusion.

[4] Galea G, Gillon J, Urbaniak SJ, Ribbons CA (1996). Study on medical donor deferrals at sessions. Transfus Med.

[5] Talonu T (1983). Causes of volunteer blood donor rejection in Papua New Guinea. P N G Med J.

[6] Chourdary RK, Gupta D, Gupta RK (1995). Analysis of donor-deferral pattern in a voluntary blood donor population. Transfus Med.

[7] Blumberg N, Shah I, Hoagland J, Shirer L, Katz AJ (1982). Evaluation of individuals deferred from blood donation for medical reasons. Vox Sang.

[8] Ranveet Kaur, Sabita Basu, Neelam Marwaha (2002). A Reappraisal of underrlysing causes in donor deferral. Ann Natl Acad Med Sci.

[9] Farrales FB, Stevenson AR, Bayer WL (1997). Causes of disqualification in a volunteer blood donor population. Transfusion.

[10] Garry PJ, VanderJagt DJ, Wayne SJ, Koehler KH, Rhyne RL, Simon TL (1991). A prospective study of blood donations in healthy elderly persons. Transfusions.

[11] Custer B, Johnson ES, Sullivan SD, Hazlet TK, Ramsey SD, Hirschler NV (2004). Quantifying losses to the donated blood supply due to donor deferral and miscollection. Transfusion.

[12] Halperin D, Baetens J, Newman B (1998). The effect of short-term, temporary deferral on future blood donations. Transfusion.

[13] Ali AM, Goldsmith CH, McAvoy AT, Ali MA, Blajchman MA (1989). A prospective study evaluating the lowering of hemoglobin standards for blood donors. Transfusion.

[14] Nishioka Ade A, Gyorkos TW, Maclean JD (2002). Tattoos and transfusion-transmitted disease risk: Implications for the screening of blood donors in Brazil. Braz J Infect Dis.

[15] Urwijitarron Y, Barusrux S, Romphruk A, Puapairoj C, Pakote L (1996). Reducing the risk of HIV transmission through blood transfusion by donor self-deferral. Southeast Asian J Trop Med Public Health.

[16] Murphy EL, David Connor J, McEvoy P, Hirschler N, Busch MP, Roberts P (2004). Estimating blood donor loss due to the variant CJD travel deferral. Transfusion.

[17] Domen RE, Grewal ID, Hirschler NV, Hoeltge GA (1997). An evaluation of the need for shared blood donor deferral registries. Int J Qual Health Care.

[18] Tomasulo PA, Anderson AJ, Paluso MB, Gutschenritter MA, Aster RH (1980). A study of criteria for blood donor deferral. Transfusion.

[19] Newman BH (2004). Whole-blood donation: Blood donor suitability and adverse events. Curr Hematol Rep.

